# Poverty, childcare responsibilities, and stigma hinder adolescent mothers from returning to school in a low-income urban informal settlement in Kenya

**DOI:** 10.1371/journal.pone.0307532

**Published:** 2024-07-22

**Authors:** Sheila Mukabana, Benta Abuya, Caroline W. Kabiru, Anthony Idowu Ajayi

**Affiliations:** Sexual Reproductive Maternal Newborn Child and Adolescent Health Unit, African Population and Health Research Center, Nairobi, Kenya; Sefako Makgatho Health Sciences University, SOUTH AFRICA

## Abstract

**Background:**

While a few studies have examined barriers to school re-entry among adolescent mothers, studies focusing on the experiences of girls in low-income informal settlements are scarce. We examined the factors that hindered parenting girls living in a resource-constrained urban setting from re-enrolling in school.

**Study setting:**

We conducted the study in Korogocho, a low-income urban informal settlement in Nairobi, Kenya.

**Methods:**

Barriers to school re-entry were documented through inductive thematic analysis of 32 in-depth interviews with pregnant and parenting adolescent girls aged 15 to 19 years (N = 22), parents/guardians (N = 10), and 10 key informant interviews with teachers (N = 4), and community leaders (N = 6).

**Results:**

Interviewed girls blamed their being out of school on their childcare responsibilities, poverty, stigmatizing and discriminatory attitudes from students and teachers, and withdrawal of parental support. While parents, teachers, and community leaders agreed that poverty and lack of childcare support hindered parenting girls from returning to school, they contended that robust support systems encompassing childcare and financial support, and less hostile school environments constituted facilitators of school re-entry among parenting adolescents.

**Conclusion:**

While the 2020 National Guidelines for School Re-entry in Kenya seek to deter the exclusion of adolescent mothers from education thereby ensuring retention, transition and completion at all basic education levels, the findings underscore the need for programs that ensure that pregnant and parenting adolescents have the requisite financial, material, and childcare support to facilitate their retention or re-enrollment in school in line with the Guidelines. School administrators and the Ministry of Education should develop and implement interventions that make the school environment less hostile for parenting girls.

## Introduction

Early childbearing has adverse effects on girls’ educational attainment. Decades of research have shown that once girls become pregnant, they voluntarily or involuntarily drop out of school and rarely return [1–4]. While adolescent mothers may want to return to school, limited support and opportunities may mean that they are instead forced to enter into marital or non-marital sexual unions [5], which may predispose them to repeat pregnancy and a lifetime of poverty [6, 7]. Adolescent mothers may also resort to engaging in income-generating activities, usually involving domestic and menial work [8] to provide for themselves and their babies. Exclusion of girls from education often results in their marginalization in the labor market as they lack negotiating power and lucrative skills compared to their educated counterparts [9].

Studies show that parenting adolescents hardly complete a full cycle of basic education [2–4] because of several barriers. First, their inability to combine childcare and school responsibilities, and financial constraints arising from poor economic backgrounds hinder them from re-entry into school [10]. Second, adolescent mothers fear being ridiculed, bullied, and stigmatized by their schoolmates, teachers, parents, and society [4, 11, 12]. Third, insurmountable pressure emanating from childcare responsibilities, financial struggles, family criticism, and societal discrimination also pushes out-of-school adolescent mothers into mental distress [13], limiting their ability to complete school.

Even though the majority of adolescent mothers do not return to school, few do, given an enabling environment that supports school readmission. Baa-Poku [14] highlights that childcare support, financial assistance, and reduced stigmatization influence adolescent mothers to continue with their education. In sub-Saharan Africa, school re-entry policies and guidelines are geared towards ensuring retention, transition, and completion [15–17] of vulnerable learners including adolescent mothers. In Kenya, the 2020 National Guidelines for School Re-entry stipulate that learners who became pregnant while in school should be re-admitted unconditionally in the same class/grade they were in before dropping out at the start of every school calendar, and in the learner’s previous school or an alternative school of choice [15]. This policy framework seeks to deter the exclusion of adolescent mothers from education thereby ensuring retention, transition, and completion at all basic education levels in line with Sustainable Development Goal (SDG) 4—Inclusive education. Despite the re-entry provisions, studies in Kenya [3, 18] indicate that adolescent girls who drop out of school due to early childbearing rarely return after delivery. Few studies have examined barriers to school re-entry in Kenya. Onyango et al. [3] revealed that retrogressive cultural norms including patriarchy, gender preference, and stigmatizing attitudes undermine school re-entry of adolescent mothers in Kenya. These findings align with previous work from Kenya [19], which found that teachers considered school re-enrollment of adolescent mothers a taboo fearing it may set a negative example for other students. Moreover, Tarus [20] found that the re-entry policy in Kenya was not clearly defined and enforced in schools.

While a few studies have explored barriers to school re-entry in Kenya, none have focused on the experiences of girls in low informal settlements, who often have to contend with intersectional vulnerabilities. Low-income urban settlements are often characterized by poor service delivery, multidimensional poverty, interpersonal violence, gender inequality, and chronic unemployment [21]. For school re-entry policies to adequately meet the needs of parenting learners, they must be evidence-based and address the needs of girls with intersectional vulnerabilities like those in spiraling low-income urban informal settlements. This study drew data from diverse categories of participants to examine barriers and facilitators of school re-entry among parenting adolescent girls in a low-income urban informal settlement.

## Methods

### Study design

Data analyzed in this paper were drawn from qualitative data of a larger concurrent equal status mixed-methods study that documented the lived experiences of pregnant and parenting adolescents in Korogocho, Nairobi, Kenya. This paper adopted an explanatory qualitative design to provide detailed evidence and explanations on the barriers and facilitators of school re-entry among adolescent mothers. The study’s goal to explain why most adolescent mothers are out of school despite Kenya’s school re-entry policy, makes explanatory qualitative design the most suitable for our study.

### Study setting

Korogocho is the fourth largest urban informal settlement in Nairobi. It is populated mostly by casual workers and is characterized by poor infrastructure, overcrowding, high crime rates, interpersonal violence, poverty, unemployment, and drug abuse [22, 23]. Girls in the study setting face intersectional vulnerability and are prone to early and unintended pregnancy [23], which makes it a suitable setting for this study. The settlement consists of nine villages with different socioeconomic, ethnic, religious, and cultural compositions.

### Positionality statement

The authors of this paper are researchers from sub-Saharan Africa, specifically based in East Africa. We have experience in conducting qualitative research among adolescent populations and other participant groups. The first author has a master’s degree in Development Studies, and has previously conducted research on social and economic development across different counties in Kenya. The second author has a doctoral degree in Comparative and International Education, and has extensive experiences conducting research on the intersections between gender and education. The third and fourth authors have doctoral degrees in Health Promotion and Sociology, respectively, and have extensive experience in researching drivers and consequences of adolescent pregnancy in Kenya and beyond. Our familiarity with the topic and the study setting makes us uniquely capable of collecting quality data and contextually analyzing barriers hindering girls from returning to school while ensuring an adequate level of anonymity, privacy, confidentiality, respect, and beneficence.

### Participant sample and sampling strategy

We drew from diverse categories of participants to allow for an in-depth and holistic understanding of the barriers and facilitators of school re-entry in urban informal settlements. Participants in this study include pregnant or parenting adolescent girls aged 15 to 19 who had lived in Korogocho for not less than 6 months at the time of data collection, parents/guardians, teachers, and community leaders. Participants were purposively sampled from 31^st^ October to 4^th^ November in 2022 to take part in the study, based on their extensive knowledge of early and unintended pregnancies among adolescent girls in the community. In-depth interviews (IDIs) were conducted with two pregnant adolescent girls, and 20 adolescent mothers. In-depth interview is the most suitable method to elicit information relevant to the study’s objective of in-depth understanding of girls’ lived experiences. The adolescent girls were identified by a local community-based organization (CBO) that has worked with young people in the community for over two decades. Our need to include a diverse group of pregnant and parenting adolescents (younger and older adolescents selected from all villages in the study setting) required working with individuals with good knowledge of the community. We provided the inclusion criteria to the CBO, and they visited households in each of the villages to identify girls, and parents who met the inclusion criteria. The CBO scheduled interview appointments in private spaces within the community, where our research assistants conducted the interviews. Ten IDIs were conducted with parents/guardians. Parents were recruited with the help of a CBO working in the community for over 20 years and programming for young girls. We recruited 10 parents because their experiences were similar and continuing with the interview did not reveal new information, that is there was saturation. Similarly, key informant interviews (KIIs) were conducted with four teachers and six community leaders selected with the help of a CBO known to the community. We interview only four teachers and six community leaders because their experiences were similar and continuing with the interviews did not reveal new information.

### Data collection procedures

Five research assistants with at least a bachelor’s degree were trained in research ethics, the interview guide, and conducting qualitative interviews, including building rapport and active listening. All five of the research assistants have previously conducted qualitative interviews with young people in rural and urban settings. Their prior experience of conducting qualitative interviews in the study setting enabled them to connect with the community and the study participants. Two of the five research assistants had lived experience as parenting adolescents. Before data collection in November 2022, interview guides for all categories of research participants were piloted to ensure they were fit for purpose. Piloting of the tools also helped the research assistants to familiarize themselves with the tool. The interview guides were developed in English, and then translated into Kiswahili to allow for the use of either language. Both IDIs and KIIs covered participants’ socio-demographic characteristics, and school re-entry barriers and facilitators. Prior to interviewing participants, written individual consent was obtained from participants aged 18 or older, whereas, written parental consent and participant assent was sought for minors. IDIs and KIIs were conducted face-to-face for approximately 1 hour 30 minutes and were tape-recorded with participants’ permission. Field notes were also taken to provide additional context.

### Data management and analysis

Audio-recordings were uploaded into a secured Google Drive folder. The folder was only accessible to research team members to ensure data security and confidentiality. Bilingual research assistants translated and transcribed the recordings into English. Inductive thematic analysis technique was employed in analyzing the data. We adopted the inductive analytical approach to identify emerging themes and patterns related to school re-entry from the qualitative data [24]. Coding was done by the first author using Microsoft Word 2021 after a reflexive reading of the transcripts. Coding involved a systematic process of generating codes to identify the emerging themes of school re-entry barriers and facilitators [24]. Emerging codes were discussed with the last author and agreed upon. The codes were organized into themes that directly answered the study objectives.

### Ethical considerations

The study protocol was reviewed and approved by the African Population and Health Research Center (APHRC) internal ethics review committee and the AMREF Ethical Scientific Review Committee (ESRC). A research permit, reference number NACOSTI/P/22/21146, was also issued by the Kenya National Commission for Science, Technology and Innovation (NACOSTI). Before fieldwork, community entry meetings were held in Korogocho to ensure acceptance and support of the study by community members. Written parental consent and participant assent for minors were obtained. Similarly, written individual consent was sought from participants aged 18 or older. Participants were allowed to withdraw from the study in part or entirely at any given time. Interviews were administered in confidentiality and at a private space provided by the local community-based organization partner. Permission was sought from the participants before audio-recording the interviews. Further, all study participants received reimbursements of 300 Kenyan shillings (~ USD 2.20) for time loss due to participating in the study. To verify the ages of the adolescent participants, individual documents such as identity cards (IDs), birth certificates, and clinic/hospital cards were scrutinized.

## Results

### Socio-demographic characteristics of the pregnant and parenting adolescent girls

Of the 22 pregnant and parenting adolescent girls who were interviewed, only 10 were aware of the school re-entry guidelines, and eight expressed a desire to return to school. Most pregnant and parenting adolescent girls (n = 14/22) who did not intend to return to school preferred to pursue vocational training such as hairdressing and catering. While some had decided on the training to pursue, others had not yet decided. Their desire to pursue vocational training stemmed from their immediate needs for money, fear of hostile school environment, and the flexibility vocational training centers offer when it comes to childcare. Some, however, desired to find menial jobs or receive support in starting up a business. The socio-demographic characteristics of the pregnant and parenting adolescent girls who participated in the study are presented in Table 1.

**Table 1:** Socio-demographic characteristics of the pregnant and parenting adolescent girls.

Participant socio-demographics	Number (N = 22)
**Age**	
15 years	1
16 years	2
17 years	6
18 years	5
19 years	8
**Highest education qualification attained**	
Dropped out of primary school	5
Completed primary education	3
Dropped out of secondary school	12
Completed secondary education	1
Still in secondary school	1
**Relationship status at the time of the interview**	
Single	20
Married/cohabiting	2
**Who they live with**	
Parents/relatives	18
Boyfriend	2
Alone	1
Female friend	1
**Employment status**	
Unemployed	15
Part-time employed (i.e., domestic or casual work)	5
Self-employed (i.e., selling shoes, bags, hairdressing)	2
**Aware of the school re-entry policy**	
Yes	10
No	12
**Intended to return to school**	
Yes	8
No (preferred vocational training e.g., hairdressing, catering)	14
**Number of children**	
One	20
None (were pregnant)	2

We present the results under two themes: 1) barriers of school re-entry, and 2) facilitators of school re-entry, in line with the study objectives. The sub-themes that emerged from analyzing barriers to school re-entry among out-of-school pregnant and parenting adolescent girls were: childcare responsibilities, poverty, stigma and discrimination, and withdrawal of parental support. While for facilitators of school re-entry sub-themes include: desire for a better future, robust support system, and engaging teachers and students to make the school environment less hostile for parenting girls.

### Barriers to school re-entry

Out-of-school pregnant and parenting adolescent girls mentioned childcare responsibilities, poverty, stigmatizing attitudes from students and teachers, and withdrawal of parental support as the key barriers to school re-entry. We describe how each of these factors hinders girls from returning to school in the following themes.

#### Childcare responsibilities

We found that adolescent mothers are the primary caregivers for their babies, a responsibility that made it challenging for them to return to school. Most of those we interviewed stayed at home to look after their babies, while a few juggled between work—usually domestic work—and childcare. To return to school, adolescent mothers reported that they would need a childminder. However, they lacked the money to pay for one. Most of the girls interviewed resided with their parents who supported them and their babies. While these parents would like to care for their grandchildren so that their daughters could return to school, they needed to work to provide for their family, including the new addition to the family. Wages from their precarious jobs were too limited to support the cost of daycare for their grandchildren and as a result, adolescent mothers sometimes had to work to augment the cost of running the home. Participants recounted situations that informed their decision to forego schooling:

“I would like to (return to school) … but if I am to go back to school, there is no one to take care of my baby. If it were my mum, she would go to work, my dad also goes to work, and my younger sister would be in school. Plus, my baby is still breastfeeding, and I do not have money to pay for daycare services, so currently I cannot do it.” (IDI participant 16, parenting girl, 17-year-old).

One parenting girl who had returned to school recounted that because her mum went out to look for work, she would sometimes go to school with her baby. When the baby became sick, she would stay at home until he got better. She recalled that they often lack money to take the baby to hospital. Since there was no one to look after her baby while she was in school, and because of the financial difficulties facing the family, she eventually decided to forego school:

“There is no one to care for the baby because my mum goes out to look for work, or other means of surviving. Sometimes I have to go to school with the baby… We also lack money to take the baby to hospital when he falls sick, so I have to stay back at home until he gets better.” (IDI participant 19, parenting girl, 19-year-old).

When asked about school re-entry, parenting girls often worriedly asked themselves, ‘what will happen to my baby?’ This question often disturbed them because their desire to return to school appeared unrealistic given their responsibility for their babies’ welfare, including safety and feeding. Interviewees contended that if they returned to school, they would worry about their babies’ welfare in daycare centers, including if they lacked milk or cried a lot. They doubted if they would be able to concentrate in class if they left their babies with strangers. One interviewee explained that concern for her baby’s welfare made her skeptical about school re-entry:

“You start thinking of how the baby is. You’ve taken them to daycare, you don’t know if the baby has eaten or not. At the same time, the milk is coming out. If you ask for permission to go breastfeed, it’s also an issue.” (IDI participant 2, parenting girl, 16-year-old).

Girls believed that the responsibility of raising their babies solely rested with them, and they did not foresee their parents taking it up. They believed they needed to make money to provide for their babies’ needs, including their education. Some saw their immediate priority as finding a job so they could provide for their children as narrated by one adolescent mother:

“I don’t think I can go back to school because when I had the desire to go to school, I never got a chance. I might go back, study, and even finish school but now, how about my child? Would she just suffer as I study? You know, it’s not like my mother will raise my child until she is all grown. She is a bit big and I can leave her with my mother and go get a job so that I can be able to take care of myself… My mother told me that she would help me raise my child in the meantime and that I should look for something that I could do to help her. So, that is why I am saying that I cannot go back to school. I need to help my child.” (IDI participant 14, parenting girl, 19-year-old).

Interviews with key informants corroborated girls’ views. There was consensus among key informants that childcare responsibilities hinder parenting girls from returning to school. Key informants such as teachers and community leaders recounted their futile efforts in getting parenting girls back to school and believed that the lack of progress was due to a lack of childcare support. One community leader recounted:

“We talk with the parents but if they refuse completely, there is nothing we can do. We tell the girl to go to Miss Koch or LVCT [Non-governmental organizations operating in the community] and see if they can help, but they also find it difficult. If they are asked to go back to school, they do not know who to leave their baby with.” (KII participant, community leader 6).

“The girl has no one to take care of her baby, and being a human being, she can’t just let her be left that way. This situation has kept so many girls out of school who would otherwise have come back, finish their studies, and become something better in this society.” (KII participant, teacher 1).

“When she goes back to school, she needs someone to take care of the baby. The parents also have to help feed the baby, though they don’t have that time.” (KII participant, community leader 4).

#### Poverty

The pregnant and parenting girls interviewed blamed their being out of school on their lack of money, and other materials needed to support their education including school uniforms. One 17-year-old interviewee stated: ‘If I say that I want to go back to school, my mother cannot afford to pay for my schooling.’ (IDI participant 10, parenting girl, 17-year-old). Girls also mentioned that they needed to work for money to be able to provide for babies, at the expense of school re-enrollment as alluded to by one interviewee:

“I mean, this child comes with a lot of expenses like diapers, they also have to eat in the morning, by 1 pm and maybe 5 or 6 pm, as well as at night. So, you do your math. Your child is used to eating like that and now you cannot provide because you are in school. So you become stressed thinking about what they ate, or what they wore. At times you do not even have soap to wash their clothes, I mean it’s a lot of things.” (IDI participant 13, parenting girl, 19-year-old).

Another interviewee stated: ‘Now that I have a baby that I am taking care of, I do not want to leave all the burden to my mum… I mostly wash other people’s clothes for pay… That is the only available job or working in a bakery.’ (IDI participant 16, parenting girl, 17-year-old).

Their mothers, most of whom had separated or divorced from their spouses, also found it difficult to financially support their daughters’ education. Interviews with mothers of pregnant and parenting adolescents revealed that lack of money was a major reason they were unable to support their daughters’ return to school. Mothers maintained that their meager resources could only settle their house rent and provide food for their families. While they desired that their daughters return to school, their lack of money meant they could only hope that help would come from somewhere else to pay for their girls’ school re-enrollment. The excerpts below paint a picture of sentiments shared by parents:

“I don’t think I have the money… where there is a good income, you can tell her to go back to school and study but, it’s very difficult if you do not have an alternative. If you get a little, you sort out rent and food. You see, it’s very difficult for me to tell her to go back to school. However, if I can get someone who can take her back to school so that she can study and finish, I will be very grateful to God.” (IDI participant, female parent 1).

“She wants to go to another school. I told her I didn’t have the money but when God provides, she will surely go. If she gets someone to send her to school, fine. As for me, I cannot even afford to buy food so it will be difficult for me to send her to school.” (IDI participant, female parent 2).

Interviews with key informants underscore poverty as a major barrier to school re-entry among parenting girls. Key informants blamed adolescent pregnancy in the community on poverty and maintained that parenting girls are unable to return to school because they lack the financial resources required to pay for books, fees, food, uniforms, and other materials:

“If one sees that even if they were to go back to school, their fees won’t be paid and they may even fail to get food. As such, they find every reason not to go back” (KII participant, community leader 1).

“Financial situations prevent young mothers from returning to school… School fees and other needs like sanitary towels.” (KII participant, community leader 4).

#### Stigma and discrimination

Despite the school re-entry policy permitting girls’ return to class after delivery, education continuation remained uncertain because girls feared being stigmatized and discriminated against by their counterparts, teachers, and community. As an adolescent mother put it: ‘I was afraid of what people would say. How will they look at me? Would they see me as a student or just a mother?’ (IDI participant 11, parenting girl, 19-year-old). Girls’ fear of stigma and discrimination is not unfounded but mostly based on their experiences as pregnant girls in school, or as parenting girls who returned to school. A few girls attempted to remain in school as long as possible and up to six months into the pregnancy. However, the school environment became hostile for them. Some recounted how students bullied and taunted them. While some just gossiped about them, others avoided them, preferring not to sit close to them. One interviewee recounted: ‘Everyone was saying, this girl is pregnant and even some weren’t talking to me. They said if they sit with me, they will also become pregnant. I just used to sit with one of my friends…’ (IDI participant 1, parenting girl, 15-year-old).

In some cases, it was the bullying that led pregnant girls to drop out of school. Bullying and victimization were not all done by students, but also by teachers. A case of a parenting girl who returned to school after her mother persuaded her to return illustrates the experiences of girls who return to school. She recounted being constantly abused verbally by the deputy principal and often in front of other students. The deputy principal would call her lazy and question why she got pregnant while in school. She narrated how the verbal attack affected her mentally resulting in her grades dropping, and she decided to quit school. She recounted her ordeal:

“She would tell me that she knew it was a must I would get pregnant because I am a very dirty girl. It was a must for me to get pregnant while still in school [Emphasis added]. The experience made me want to drop out of school, but I thought about it and concluded that it was my life. Even though I had messed it up, it was better for me to just pick up and continue with it.” (IDI participant 8, pregnant girl, 19-year-old).

Key informants affirmed that parenting adolescents are not banned from school but they are taunted by fellow students. Informants stated that parenting girls are labeled as “Masa” [meaning mother], a term used to separate them from regular students. They believed that it is the shame and embarrassment resulting from being taunted constantly that made parenting adolescents fail to return to school:

“The challenge that these young mothers go through is being mocked by others, hence rendering it difficult for them to continue with their education. In most cases, even if they were registered for their Form Four exams, most of them prefer to stay at home and only show up for exams. In such cases, most of them do not do well.” (KII participant, community leader 2).

#### Withdrawal of parental support

Frustrations, emanating from disappointment and broken trust, bred rejection and withdrawal of parental support for the pregnant and parenting girls. Community leaders and teachers observed that parents were unwilling to take responsibility for their daughters’ education because they felt that responsibility should shift to their girl’s partners. Some parents viewed them as mature individuals who needed not to study. This view was echoed by key informants:

“The parents say they would not pay school fees for a woman who has had a child, or is pregnant and has a boyfriend. They are usually told, ‘Go to the person you agreed to have sex with to support you’. …because of that, their education ends there as well as support in terms of feeding.” (KII participant, community leader 2).

“Parents refuse to pay school fees, saying they cannot educate somebody’s wife. They can’t waste money on a fellow woman…” (KII participant, teacher 2).

Parents’ limited income made them decide that their daughter’s education was no longer a priority. They considered their daughter as undeserving of a second chance at education, and instead expected that their daughters should focus on their own children’s education. One parenting girl recalled the conversation she had with her father: ‘I was willing to go back to school even though I was pregnant… I accepted and even asked for forgiveness from my father, but he refused and said there was no way I could go back to school.’ (IDI participant 12, pregnant girl, 18-year-old). A key informant also narrated how a bright student got pregnant and tried unsuccessfully to persuade her brothers to facilitate her return to school:

“There is a case I handled of a girl called… She was in secondary school, got pregnant, and had to stay at home. She was a very bright student and wanted to go back to school, but now there was no one to pay her fees. She went to the brothers, but they told her that the little they had, had been to given another person since she already wasted her chance.” (KII participant, community leader 4).

### Facilitators of school re-entry

Study participants expressed their views on strategies to facilitate school re-entry of parenting adolescent girls who returned to school, and opined on factors that enabled them. Results from our analysis of enablers of school re-entry are discussed under the following themes: desire for a better future, robust support system, and engaging teachers and students to make the school environment less hostile for parenting girls.

#### Desire for a better future

Girls who returned to school were resilient in the face of hostile school environment. They desired to change their circumstances, characterized by socioeconomic struggles in overcrowded and impoverished neighborhoods, through education. They strongly believed that achieving their dreams would help transform their lives, and that of their children, family, and the community. Interviewees confidently expressed their yearning for a rewarding future. When asked if she decided to go back to school, or if it was her mother who encouraged her, one parenting girl who had returned to school reiterated:

“I decided to go back so that I can finish Form 4 (secondary education) … I could not miss the exam… I wanted to get my certificate.” (IDI participant 22, parenting girl, 18-year-old).

Another one put the reason for her return to school down to her strong desire to accomplish her dream of becoming a journalist:

“I just want to achieve my dreams… I wanted to be a journalist… or a radio presenter. So, I usually feel like I have not yet achieved my dreams, and that makes me feel very bad that I just wasted my time, and so I want to recover that by going back to school.” (IDI participant 17, pregnant girl, 18-year-old).

#### Robust support system

A recurring theme that emerged from the study findings was childcare and financial support are key to facilitate parenting girls’ return to school. Interviewees reiterated that childcare assistance is a key prerequisite for school re-entry, continuation, and successful completion. The two interviewed parenting girls who had returned to school emphasized that that was possible because their mothers, friends and grandmothers provided childcare support when in school:

“My mum takes care of my child every day… I am in school… I get to stay with the baby on Saturdays. If my mum is not there and I’m also not there, maybe I am in school and the child needs to go to the clinic, my friends take the child there for me.” (IDI participant 22, parenting girl, 18 years old).

“My grandmother takes care of my child… I also do a little bit, but when I go school, I leave the baby with her.” (IDI participant 21, parenting girl, 17 years old).

While childcare assistance could be offered by family or friends of the adolescent mothers, a community leader suggested daycare centers as possible alternatives when parenting girls are in school:

“There should also be a daycare center where they can leave their babies. After school, they can pick their babies…” (KII participant, community leader 2).

Study participants also highlighted the importance of financial support. A parenting girl reported: ‘Whenever I get someone who would take me back to school, even in Form 2 (secondary level), I am willing to go back and study. I can never refuse to go back to school.’ (IDI participant 16, parenting girl, 17-year-old). This view was supported by another parent’s account: ‘They should be supported with things like school fees so they can go back to school… school fees, food, sanitary towels.’ (IDI participant, female parent 3).

#### Engaging teachers and students to make the school environment less hostile for parenting girls

Study participants maintained that there is a need to make the school environment less hostile for parenting girls. Acceptance in school would mean they are not stigmatized by teachers and other learners. One parenting adolescent reiterated what she considered would facilitate parenting girls’ school entry:

“…there shouldn’t be those people who keep on saying, ‘Those that got pregnant should stay on their side’. Also, teachers in school shouldn’t be telling others not to sit next to those who have given birth.” (IDI participant 3, parenting girl, 16-year-old).

Teachers also alluded to acceptance in school as a facilitator of school re-entry. They argued that adolescent mothers should receive respectful treatment, guidance and counselling support, and other learners should be sensitized through clubs against discriminating against adolescent mothers to facilitate school re-entry:

“We teachers give them preferential treatment. She can come to school, yet yesternight she never had time to rest because the baby was crying. You won’t push her the way you push the rest of the students.” (KII participant, teacher 3).

“We should create our sensitization measures, perhaps create some clubs. It would also be great if we get support from some NGOs to help us with talking to the girls.” (KII participant, teacher 4).

## Discussion

This study assessed barriers and facilitators of school re-entry among parenting girls in an urban informal settlement in Kenya. We found that childcare responsibilities, poverty, stigma and discrimination, and withdrawal of parental support were the key barriers associated with school re-entry. On the other hand, desire for a better future, robust support systems, and engaging teachers and students to make schools less hostile for parenting girls are perceived enablers of re-entry.

Consistent with previous studies in other settings [4, 25], our study shows that childcare responsibilities hinder parenting girls from re-enrolling in school. Childcare roles were mainly performed by the girls since their parents were preoccupied with work, and they lacked the money to take their children to daycare centers. We also found that childcare support was emphasized as a facilitator of school re-entry, as shown in other studies [14, 26]. Because they lacked childcare support, many parenting adolescents abandoned their educational goals to focus on caring for their babies. They often turned to domestic or menial jobs to raise money to meet their babies’ needs. Similarly, a study in South Africa found that out-of-school adolescent mothers were engaged in income-generating activities [8]. Collectively, the findings from this and other studies highlight the need for programs that increase access to quality childcare for parenting adolescents living in resource-constrained settings.

One important finding of this study is that poverty hinders parenting girls from re-enrolling in school, a finding that has been reported in other settings in Kenya [25, 27], Burkina Faso [28], and Malawi [1]. Both parents and parenting girls alluded to lacking money to pursue school re-enrollment, and consider finding money to survive and care for their children to be more urgent. In low-income informal settlements, like Korogocho, parents are mostly employed in menial or casual jobs, if at all employed. The meager income earned hardly caters to school-related expenses. While primary and secondary education are free in Kenya, there are additional costs associated with schooling including transportation, uniforms, meals, and textbooks. Poverty also makes parents withdraw their support for their girls’ education preferring to focus their resources on other priorities. Our study shows that financial support and sponsorship is considered to be a key facilitator of school re-entry. This result is in line with evidence from Western Kenya [3], which suggested that economically endowed parents are likely to support school re-entry compared to poor parents. These findings point to the need for social protection programs to consider pregnant and parenting adolescents as a sub-population that may need special considerations.

Previous research has shown that discriminatory and stigmatizing attitudes in schools hinder re-entry [11, 29, 30]. Consistent with Malatji et al. [12], we found that negative comments coupled with ridicule, mockery, and gossip perpetrated by students and teachers hinder parenting girls from re-enrollment in learning institutions, since they feel shy and discouraged. Many girls have not attempted to return to school despite expressing their willingness to return to school because of the fear of being stigmatized and discriminated against. Parenting girls perceived the school environment to be hostile and unwelcoming to pregnant and parenting girls. However, a study conducted in South Africa reported contrary findings [31] highlighting teachers, though few, are protective against judgmental and stigmatizing attitudes in schools by providing counselling. Engaging school administrators, teachers, and students to make the school environment welcoming for parenting adolescents is an important strategy to facilitate school re-entry. Teachers in the school setting, and social workers in communities can provide counselling services to adolescent mothers to ensure positive behavior, psychological wellness, and academic support.

This research also shows the important role that parents or caregivers and other family members play in hindering or facilitating school re-entry for pregnant and parenting adolescents. Similarly, a study in Western Kenya [32] highlighted the critical role of parental support in facilitating school re-entry. Interventions to improve school re-entry or retention for pregnant and parenting adolescents should target their parents and other family members to create an enabling environment for school re-entry.

Another key finding of this study is that desire for a better future is considered a facilitator of school re-entry. Girls who returned to school, propelled by a strong desire to achieve their dreams, demonstrated resilience in the face of hostility, childcare responsibilities, and financial difficulties. A study in Kenya [25] indicates that adolescent mothers associated their life improvement and career development with education. While guidance and counselling can help build the desire for a better future, Amod et al. [33] reported that parenting adolescents rarely receive emotional support in school.

### Policy implications

Despite the National Guidelines for School Re-entry by the Kenyan government [15], adolescent mothers are reluctant to re-enroll in school because they lack financial resources, consider school environment to be hostile, and lack childcare support that would enable them to re-enroll. It is therefore pertinent that the guidelines are revised to address hostile school environment. Clear strategies should be stipulated on how to ensure safe and conducive learning environment for pregnant and parenting students. Lack of financial support could be addressed through allocating bursaries and other school requirements such as uniforms and books, to adolescent mothers dwelling in low-income informal settlements. Government social protection programs could be extended to vulnerable pregnant and parenting girls to ease their financial burden. Childcare concerns could be addressed by providing subsidized childcare for girls whose families are unable to assist with childcare roles. Further, schools need to continuously sensitize students and teachers against stigmatizing and discriminating in-school adolescent mothers. Finally, pregnant and parenting adolescents may not be aware of their right to remain in school. A tracer intervention focused on tracking and tracing of out-of-school adolescent mothers by the Ministry of Education, and talking to them on the school re-entry guidelines to promote their return to school was found to be effective in increasing school re-entry [32]. Similar interventions could be adopted at the community level to facilitate retention, transition, and completion of schooling for pregnant and parenting adolescents.

### Strengths and limitations

An important strength of this paper is the triangulation of experiences and perspectives of pregnant and parenting girls, parents, teachers, and community leaders to provide an in-depth understanding of the barriers and facilitators of school re-entry in a low-income urban informal settlement. However, the study is not without limitations. Our study was limited to an urban informal setting where there is an increased risk of early pregnancies among adolescent girls, who mostly come from impoverished households. The findings may not represent the experiences of girls from non-slum areas, or middle to high-income families.

## Conclusion

Overall, our findings reaffirm that parenting girls rarely return to school despite school re-entry provisions. We found that re-entry barriers include childcare responsibilities, poverty, stigma and discrimination, and withdrawal of parental support. School re-entry barriers limit the educational achievement and employment opportunities of adolescent mothers. The findings underscore the need for financial, material, and childcare support for parenting girls from low socioeconomic backgrounds, which are currently lacking in the school re-enrollment policy, to facilitate re-entry, retention, transition, and completion. School administrators and the Ministry of Education should also develop and implement sensitization campaigns against the stigma and discrimination of adolescent mothers in schools.
